# Mononuclear binding and catalytic activity of europium(III) and gadolinium(III) at the active site of the model metalloenzyme phosphotriesterase

**DOI:** 10.1107/S2059798324002316

**Published:** 2024-03-21

**Authors:** Callum W. Breeze, Yuji Nakano, Eleanor C. Campbell, Rebecca L. Frkic, David W. Lupton, Colin J. Jackson

**Affiliations:** a Research School of Chemistry, Australian National University, Canberra, ACT 2601, Australia; b Australian Research Council Centre of Excellence for Innovations in Peptide and Protein Science, Research School of Chemistry, Australian National University, Canberra, ACT 2601, Australia; c School of Chemistry, Monash University, Clayton, Melbourne, VIC 3800, Australia; d Australian Synchrotron, 800 Blackburn Road, Clayton, Melbourne, VIC 3168, Australia; e Australian Research Council Centre of Excellence in Synthetic Biology, Research School of Chemistry, Australian National University, Canberra, ACT 2601, Australia; University of Queensland, Australia

**Keywords:** lanthanides, phosphotriesterase, anomalous scattering

## Abstract

Lanthanide ions have ideal chemical properties for catalysis, such as hard Lewis acidity, fast ligand-exchange kinetics, high coordination-number preferences and low geometric requirements for coordination. As a result, many small-molecule lanthanide catalysts have been described in the literature. Yet, despite the ability of enzymes to catalyse highly stereoselective reactions under gentle conditions, very few lanthanoenzymes have been investigated. In this work, the mononuclear binding of europium(III) and gadolinium(III) to the active site of a mutant of the model enzyme phosphotriesterase are described using X-ray crystallography at 1.78 and 1.61 Å resolution, respectively. It is also shown that despite coordinating a single non-natural metal cation, the PTE-R18 mutant is still able to maintain esterase activity.

## Abstract 2

The crystal structures of europium(III)- and gadolinium(III)-bound phosphotriesterase are presented along with esterase activity data for the lanthanide-bound enzymes.

## 1. Introduction

### 1.1. Lanthanides

The lanthanide elements (Ln), with atomic numbers 57–71, also referred to as rare-earth elements, are relatively naturally abundant metals which have found uses in many modern technologies (Cotruvo, 2019 ▸; De Simone *et al.*, 2018 ▸). These metals are stable in their 3+ oxidation state [Ln(III)] under aqueous conditions, losing their 5*d* and 6*s* electrons, but maintaining their 4*f* electrons with the electronic configuration [Xe]4*f* ^*n*^ [where *n* = 0–14 from La(III) to Lu(III)] (Cotruvo, 2019 ▸; Daumann, 2019 ▸; Bünzli, 2006 ▸). Cerium is also stable in the 4+ oxidation state (Franklin, 2001 ▸).

The 4*f* valence shell of Ln(III) ions is shielded by the outer 5*s* and 5*p* shells, resulting in a 4*f* shell that lies inert, deep in the cation (Bünzli, 2006 ▸; Mikami *et al.*, 2002 ▸; Evans, 2000 ▸). This buried 4*f* orbital offers poor shielding of the outer orbitals from nuclear charge, resulting in the relatively small size of the lanthanides, referred to as the ‘lanthanide contraction’ (Cotruvo, 2019 ▸). Another consequence of the buried 4*f* orbitals is a lack of orbital overlap with coordinating ligands with respect to *d*-block transition-metal coordination, resulting in organolanthanide interactions that are predominantly ionic in nature (Cotruvo, 2019 ▸; Mikami *et al.*, 2002 ▸). These ionic interactions result in little steric requirement for coordination, in stark contrast to *d*-block transition metals; thus, Ln(III) ions have a preference for coordination numbers (CNs) of 8–12 (Jahn *et al.*, 2018 ▸; Deng *et al.*, 2018 ▸; Pol *et al.*, 2014 ▸; Bünzli, 2006 ▸; Franklin, 2001 ▸; Shannon, 1976 ▸). A further consequence of the buried and diffuse 4*f* orbitals, and the predominantly ionic interactions, is that Ln(III) ions have little ligand-field splitting effect; thus, Ln(III) complexes have fast ligand-exchange kinetics (Bünzli, 2006 ▸; Franklin, 2001 ▸).

### 1.2. Lanthanides as catalysts

Ln(III) chemistry, specifically the high Lewis acidity, high charge, fast ligand-exchange kinetics, high CN, flexible coordination geometry and redox inertness, make Ln(III) ions ideal for catalysis (Cotruvo, 2019 ▸; Lim & Franklin, 2004 ▸). For example, the NPAC–La(III) complex is capable of hydrolysing the phosphodiester bonds of DNA and RNA (Baykal & Akkaya, 1998 ▸). This complex uses neutral ligands so as to not reduce the Lewis acidity of La(III) (Baykal & Akkaya, 1998 ▸). The Lewis acidity and high CN of the metal are required for catalysis to occur (Franklin, 2001 ▸; Komiyama *et al.*, 1999 ▸).

Despite ideal catalytic properties and similar size to Ca(II), it was thought that Ln(III) ions were completely absent from life due to their strong Lewis acidity resulting in low solubility at neutral pH (Peplow, 2021 ▸; Cotruvo, 2019 ▸; Daumann, 2019 ▸; Allen & Imperiali, 2010 ▸; Franklin, 2001 ▸; Firsching & Brune, 1991 ▸). Yet, in 2011 the first lanthanoenzyme was discovered in Gram-negative methylotrophic bacteria, suggesting that Ln(III) ions could play important, albeit rare, roles in biology (Fitriyanto *et al.*, 2011 ▸; Hibi *et al.*, 2011 ▸).

With their unique chemistry, it may be that Ln(III) ions could be used to augment enzyme chemistry, permitting the turnover of synthetic non-natural substrates, whilst retaining the high regioselectivity and stereoselectivity of enzymes. Indeed, haem-containing metalloenzymes have been engineered via the introduction of non-natural metals in place of iron, which has resulted in non-natural catalysis of synthetic substrates (Dydio *et al.*, 2017 ▸; Key *et al.*, 2016 ▸, 2017 ▸).

### 1.3. Lanthanides in biology

Previous proposals have suggested that lanthanoenzymes should be more common than they are, and that Ln(III) is better suited for catalysis than Ca(II), for instance (Lim & Franklin, 2004 ▸). Despite this, there are few known examples of lanthanoenzymes and lanthanoproteins.

The first natural lanthanoenzyme to be described was XoxF-MDH in 2011, a methyl dehydrogenase (MDH) containing a pyrroloquinoline quinone (PQQ) cofactor along with a Ln(III) cofactor, which catalyses the two-electron oxidation of methanol (Good *et al.*, 2020 ▸; Vu *et al.*, 2016 ▸; Nakagawa *et al.*, 2012 ▸; Fitriyanto *et al.*, 2011 ▸; Hibi *et al.*, 2011 ▸). There is also a Ca(II)-binding MDH analogue, MxaF-MDH, which catalyses the same oxidation of methanol (Chistoserdova & Lidstrom, 1997 ▸; Anthony & Zatman, 1964*a* ▸,*b* ▸). The two enzymes are very similar, differing only in an additional aspartate in the Ln(III) coordination sphere of XoxF-MDH, increasing the CN from seven to nine, which satisfies the high CN preference and stabilizes the 3+ oxidation state (Good *et al.*, 2020 ▸). As a result of the extra ligand, the Ln(III) ion has a higher binding affinity for XoxF-MDH than Ca(II) does for MxaF-MDH (Nakagawa *et al.*, 2012 ▸). The high charge density and Lewis acidity of Ln(III) with respect to Ca(II) results in XoxF-MDH having a higher catalytic efficiency than MxaF-MDH (Nakagawa *et al.*, 2012 ▸; Fitriyanto *et al.*, 2011 ▸; Hibi *et al.*, 2011 ▸). This may suggest that Ln(III) ions are indeed preferable to Ca(II) in enzymes, as was initially suggested in 2004, and indicates that the development of lanthanoenzymes would be a fruitful line of enquiry that could produce catalytically efficient enzymes (Lim & Franklin, 2004 ▸). Since this discovery, further Ln(III)-bound alcohol oxidases have been described such as ExaF, an ethanol dehydrogenase (Good *et al.*, 2016 ▸).

In 2018, an Ln(III)-binding protein was discovered called lanmodulin (LanM), which binds Ln(III) ions using EF-hand motifs (Cook *et al.*, 2019 ▸; Cotruvo *et al.*, 2018 ▸). The function of this protein has yet to be determined, but clearly indicates the potential for a greater contribution of Ln(III) ions to protein fold and function in the bacterial kingdom (Featherston & Cotruvo, 2021 ▸; Cotruvo *et al.*, 2018 ▸). Lanmodulin provides lessons in designing Ln(III) chelators, as the LanM EF-hand has an additional aspartate with respect to calmodulin EF-hands, thus satisfying the high CN preference of Ln(III) ions and the high charge, but also indicating the similarity in binding domains between the metals (Cook *et al.*, 2019 ▸; Cotruvo, 2019 ▸; Cotruvo *et al.*, 2018 ▸). Notably, this difference provides lanmodulin with higher binding affinities for Ln(III) than calmodulin has for Ca(II); these are in the micromolar range (VanScyoc *et al.*, 2002 ▸; Linse *et al.*, 1991 ▸).

To date there has been one example of an engineered lanthanoenzyme, the chimeric 33-mer metallopeptide ‘P3W’ formed by the fusion of an EF-hand motif of calmodulin and the helix–turn–helix motif of the engrailed homeodomain (Sirish & Franklin, 2002 ▸; Kim *et al.*, 2001 ▸; Welch *et al.*, 2001 ▸). The design of this chimera was achieved by superposition of the structures of calmodulin and the engrailed homeodomain, which have very similar structures. The chimera permitted DNA binding and hydrolysis of supercoiled DNA, due to the proximal positioning of the Ln(III) ion to the phosphodiester bond. Overall, these enzymes provide proof-of-principle that lanthanoenzymes can be engineered and that Ln(III) ions maintain sufficient Lewis acidity for catalysis when coordinated by negatively charged ligands.

Most work coordinating Ln(III) ions to proteins has been for the purpose of X-ray crystallography or NMR, and has been executed using synthetic organic polydentate ligands that are attached via non-natural amino acids or cysteine residues (Herath *et al.*, 2021 ▸; Loh *et al.*, 2013 ▸; Allen & Imperiali, 2010 ▸; Silvaggi *et al.*, 2007 ▸). Ln(III) ions scatter X-rays extremely well and thus can aid in solving the phase problem when solving crystal structures (Silvaggi *et al.*, 2007 ▸; Harker, 1956 ▸). Ln(III) ions are also useful for NMR studies due to their paramagnetism, which produces pseudocontact shifts in the protein of up to 40 Å from the ion; thus, tagging a protein with an Ln(III) ion provides long-range structural information (Pilla *et al.*, 2017 ▸; Yagi *et al.*, 2013 ▸; Schmitz *et al.*, 2012 ▸; Allen & Imperiali, 2010 ▸). However, for crystallography the Ln(III) ion must be well ordered in relation to the protein, and for NMR Ln(III) mobility results in decreased information; therefore, a high-affinity Ln(III)-binding tag is required (Allen & Imperiali, 2010 ▸). Hence, the Ln(III)-binding tags used in protein NMR experiments have the requisite features to accommodate Ln(III) ions: a high CN and hard Lewis base ligands (Herath *et al.*, 2021 ▸; Loh *et al.*, 2013 ▸).

Development of lanthanoenzymes could result in biocatalysts that are capable of catalysing non-natural reactions using non-natural substrates, resulting in potentially useful products being produced at low temperatures under gentle conditions. In this work, we describe a lanthanide-bound model enzyme. Phosphotriesterase (PTE), a binuclear Zn(II) metalloenzyme from *Pseudomonas diminuta*, has previously undergone directed evolution to change its activity from a phosphotriesterase to an esterase of 2-naphthyl hexanoate (2NH); the round 18 variant (PTE-R18) in this engineering pathway had an additional Zn(II)-binding site within the active site, indicating a loss of specificity for two divalent metals (Tokuriki *et al.*, 2012 ▸). PTE-R18 was generated from PTE by the mutations H254R, D233E, F306I, I274S, T172I, S269T, M138I, T199I, L272M, A80V, S111R, A204G, L130V, L271F, A49V, K77E, L140M and I313F (Tokuriki *et al.*, 2012 ▸). Given that PTE is naturally promiscuous for divalent metal coordination, combined with this additional loss of specificity for binding two cations, it was hypothesized that PTE-R18 could have the potential to bind unusual metals in its active site. Ln(III) ions were an interesting candidate, as their larger size compared with Zn(II) ions could result in mononuclear occupancy of the unusual ‘third’ binding site in PTE-R18. Here, we describe the coordination and affinity of these mononuclear Ln(III)–PTE-R18 complexes using X-ray crystallography and isothermal titration calorimetry, and we show that esterase activity is maintained by the enzyme.

## 2. Methods

### 2.1. Production and purification of PTE-R18

PTE-R18 was overexpressed from pETMCSI-PTE-R18 (see the supporting information). Production and purification were performed as described previously (Campbell *et al.*, 2016 ▸).

### 2.2. Formation and verification of apo PTE-R18

Zn(II) was removed from PTE-R18 by two rounds of dialysis against chelation buffer (5 m*M* HEPES, 100 m*M* NaCl, 5 m*M* 1,10-phenanthroline pH 8.0) in a 1:100 ratio through SnakeSkin Dialysis Tubing 10K MWCO 35 mm (Thermo Fisher Scientific, USA) for 24 h at 4°C. The chelator 1,10-phenanthroline was subsequently removed by four exchanges against dialysis buffer (5 m*M* HEPES, 100 m*M* NaCl pH 8.0) at a 1:100 ratio through SnakeSkin Dialysis Tubing 10K MWCO 35 mm for 8 h at 4°C. PTE-R18 retention was determined by SDS–PAGE. The formation of apo PTE-R18 was confirmed via incubation of 100 n*M* enzyme with 2.5 m*M* *p*-nitrophenyl butyrate in assay buffer [20 m*M* Tris, 100 m*M* NaCl, 2.5%(*v*/*v*) methanol pH 8.5] with the positive control containing 100 µ*M* ZnCl_2_, and analysis of the absorbance at 405 nm over 20 min at room temperature.

### 2.3. Crystallization

All proteins were buffer-exchanged into 20 m*M* HEPES, 50 m*M* NaCl pH 8.0 via centrifugation at 3000*g* and 4°C using an Amicon Ultra-15 Centrifugal Filter 10 kDa MWCO prior to crystallization. All crystals were grown at 4°C using 100 m*M* sodium cacodylate, 14%(*v*/*v*) MPD pH 6.5 as mother liquor (ML) by hanging-drop diffusion. Zn-PTE-R18 (22 mg ml^−1^) was crystallized at a 2:1 ML:protein ratio. These crystals were manually pulverized for use as a Zn seed stock.

Apo PTE-R18 (12 mg ml^−1^) was crystallized at a 1.5:1:0.5 ML:protein:Zn seed stock (1 × 10^−8^ dilution) ratio. These crystals were manually pulverized for use as an apo seed stock.

Apo PTE-R18 (10 mg ml^−1^) was incubated with 2.77 m*M* La(NO_3_)_3_, Pr(NO_3_)_3_, Sm(NO_3_)_3_, EuCl_3_ or GdCl_3_ for 30 min at 4°C to form Ln-PTE-R18. Ln-PTE-R18 (10 mg ml^−1^) was crystallized at a 1.5:1:0.5 ML:protein:apo seed stock (1 × 10^−8^ dilution) ratio. All crystals were manually looped and cryoprotected in 100 m*M* sodium cacodylate, 40%(*v*/*v*) MPD, 0.92 m*M* LnCl_3_ pH 6.5, which was immediately followed by flash-cooling in liquid nitrogen.

### 2.4. Fluorescence scans

To assess the presence of lanthanides in the crystal samples, the fluorescence emission spectrum was measured using Se *K*-edge detection with input energy 12 857.8 eV and a scan time of 30 s using BL_3ID1 on the MX2 beamline at the Australian Synchrotron (Aragão *et al.*, 2018 ▸).

### 2.5. Structure determination

Diffraction data were collected on beamline MX2 at the Australian Synchrotron (Aragão *et al.*, 2018 ▸). Data for the apo PTE-R18 structures were collected at 13 and 9.5 keV, and data for the Ln-PTE-R18 structures were collected at 9.5 keV to allow the collection of anomalous data resulting from the lanthanides. All data were collected with 70% attenuation of the beam, with detector distances of 130 mm for the apo and Eu(III)-bound structures and 150 mm for the Gd(III)-bound structure.

Data were indexed and integrated using *XDS*, followed by resolution estimation and data truncation using *AIMLESS* as implemented in *CCP*4 (Evans & Murshudov, 2013 ▸; Agirre *et al.*, 2023 ▸; Kabsch, 2010 ▸). All structures were solved by molecular replacement using *Phaser MR* as implemented within *CCP*4, using PDB entry 4e3t as the search model (Tokuriki *et al.*, 2012 ▸; Agirre *et al.*, 2023 ▸; McCoy, 2007 ▸; McCoy *et al.*, 2007 ▸). The model was iteratively refined and optimized using *phenix.refine* and *Coot* version 0.9.8.7 (Afonine *et al.*, 2012 ▸; Emsley *et al.*, 2010 ▸). *mF*_o_ − *DF*_c_ density was used to model alternative conformations, and *B* factors were determined using *phenix.refine* (Afonine *et al.*, 2012 ▸). The structures were validated using *MolProbity*. *Phenix* version 1.20.1 produced the refinement statistics (Table 1 ▸; Liebschner *et al.*, 2019 ▸; Afonine *et al.*, 2010 ▸). The finalized structures were visualized and analysed in *PyMOL* version 2.5.3 (DeLano, 2004 ▸). The coordination of metals by residue ligands was assessed using the *CheckMyMetal* server (Gucwa *et al.*, 2023 ▸; Handing *et al.*, 2018 ▸; Zheng *et al.*, 2014 ▸, 2017 ▸). CIF files for coordinates and structure factors were generated using *pdb_extract* and were validated using the wwPDB Validation System (Yang *et al.*, 2004 ▸).

### 2.6. Isothermal titration calorimetry

To determine the binding stoichiometries of Eu(III) and Gd(III) to apo PTE-R18, isothermal titration calorimetry was performed using a Nano ITC low-volume calorimeter (TA Instruments). All experiments were performed at 20°C with stirring at 200 rev min^−1^, with 23 × 2 µl serial injections of 1.5 m*M* EuCl_3_ and 2 m*M* GdCl_3_ into 238 and 360 µ*M* apo PTE-R18 (monomer concentration), respectively, with 360 s intervals.

Data were integrated using *NITPIC* version 1.2, followed by global analysis using *SEDPHAT* version 12.1 (Scheuermann & Brautigam, 2015 ▸; Zhao *et al.*, 2015 ▸). Data were analysed using the ‘A + B ↔ AB Hetero-Association’ model and the Marquardt–Levenberg equation to fit a function to the data. The data were plotted and visualized using *GUSSI*.

### 2.7. Ln(III)-PTE-R18 activity assays

To determine the activities of Eu(III)- and Gd(III)-bound PTE-R18, hydrolysis of 2NH was quantified. Assays consisted of 500 µ*M* EuCl_3_ or GdCl_3_, 50 µ*M* apo PTE-R18, 10 m*M* 2NH substrate and 5%(*v*/*v*) methanol and were performed in 20 m*M* Tris, 150 m*M* NaCl pH 7.0 or 8.0. Reactions were incubated at room temperature with mixing for 42 h. Control reactions were performed without apo PTE-R18. Reactions were treated with a 4.5× volume of acetonitrile with 2.2 m*M* 1,3,5-trimethoxybenzene, followed by filtration through a 0.22 µm filter and concentration under nitrogen gas. The formation of 2-naphthol (2N) was determined by ^1^H NMR at 400 MHz using CDCl_3_.

## 3. Results and discussion

### 3.1. Formation of apo PTE-R18

The strong chelating agent 1,10-phenanthroline was used to remove the co-purified and tightly bound Zn(II) ions to permit lanthanide binding in the active site of PTE-R18. The removal of Zn(II) was confirmed using a colorimetric assay and X-ray diffraction experiments on apo PTE-R18 crystals.

#### 3.1.1. Hydrolysis assay

**Figure 1:**
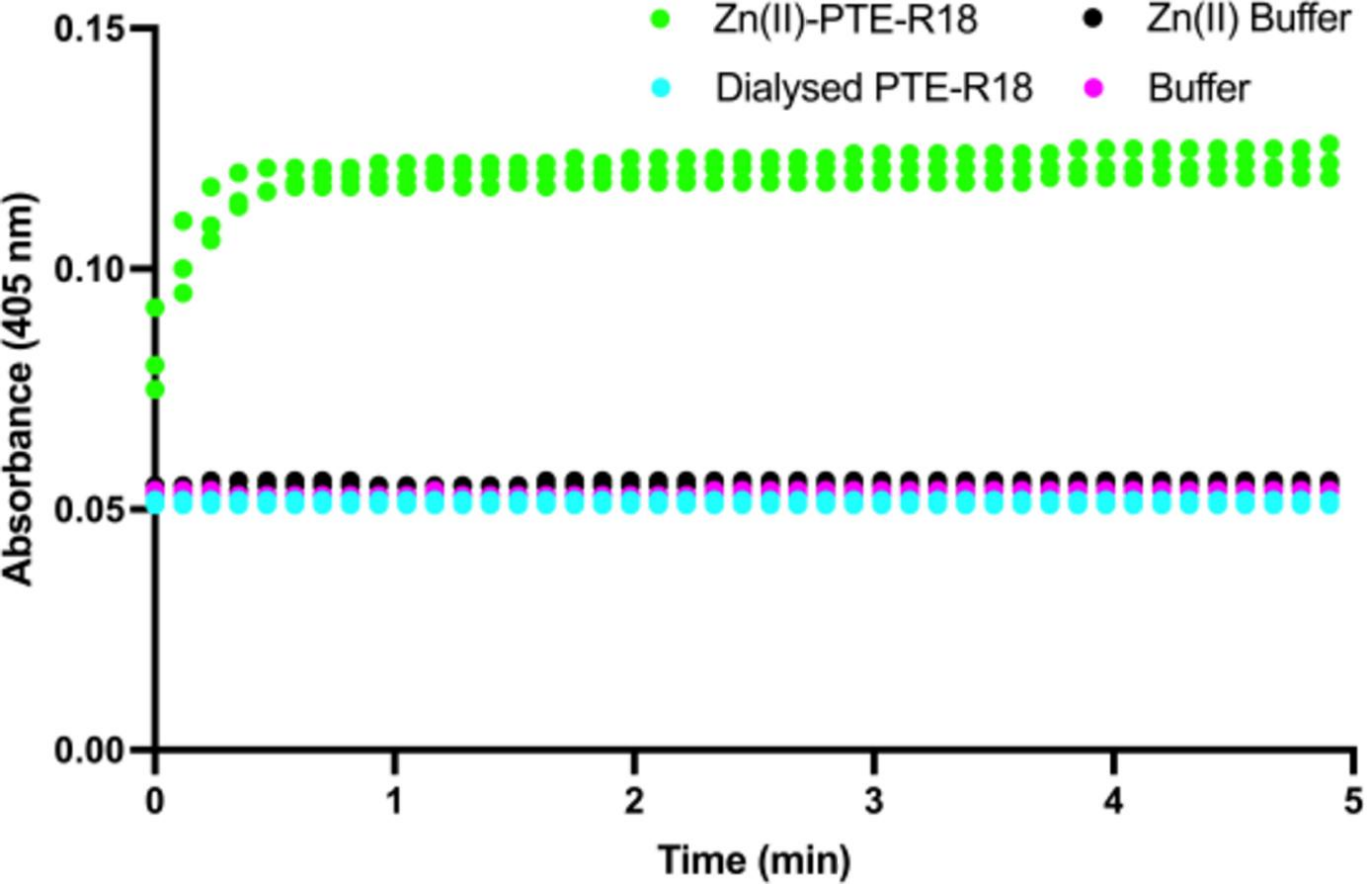
*Para*-nitrophenyl butyrate hydrolysis assay. *Para*-nitrophenol absorbance at 405 nm, measured over 5 min, with buffer consisting of 20 m*M* Tris, 100 m*M* NaCl, 2.5%(*v*/*v*) methanol pH 8.5 (magenta) and with buffer with 100 µ*M* ZnCl_2_ (black), with 100 n*M* dialysed protein (cyan) and with 100 n*M* dialysed protein and 100 µ*M* ZnCl_2_ (green) (*N* = 3).

To test for the removal of Zn(II), hydrolytic enzyme activity against *para*-nitrophenyl butyrate (pNPB) was assessed. Previous work has demonstrated the ability of PTE-R18 to rapidly hydrolyse *para*-nitrophenyl acetate, yielding a yellow product (Tokuriki *et al.*, 2012 ▸). The product of the hydrolysis reaction, *para*-nitrophenol (*para*-nitrophenolate at pH 8.5), was measured by absorbance at 405 nm (Fig. 1 ▸). The dialysed enzyme showed a complete lack of *para*-nitrophenol production, with an absorbance at 405 nm similar to those of the buffer controls, whereas the Zn(II)-supplemented apo PTE-R18 sample demonstrated a rapid increase in absorbance at 405 nm, indicating a loss of activity of the dialysed protein and thus successful Zn(II) removal. The reconstituted enzyme achieved complete hydrolysis of 2.5 m*M* pNPB after ∼30 s, resulting in an estimated *k*_cat_ of 809 ± 165 s^−1^, which is comparable to the reported *k*_cat_ for hydrolysis of the ester 2NH of 880 ± 30 s^−1^, demonstrating that the dialysed protein can be reconstituted with metal ions to give native holoenzyme activity (Kaltenbach *et al.*, 2015 ▸). Previous work has also shown that 1,10-phenanthroline is capable of chelating Zn(II) from PTE-R18 and that PTE-R18 is stable in the apo form (Tokuriki *et al.*, 2012 ▸).

#### 3.1.2. Apo PTE-R18 crystallography

**Figure 2:**
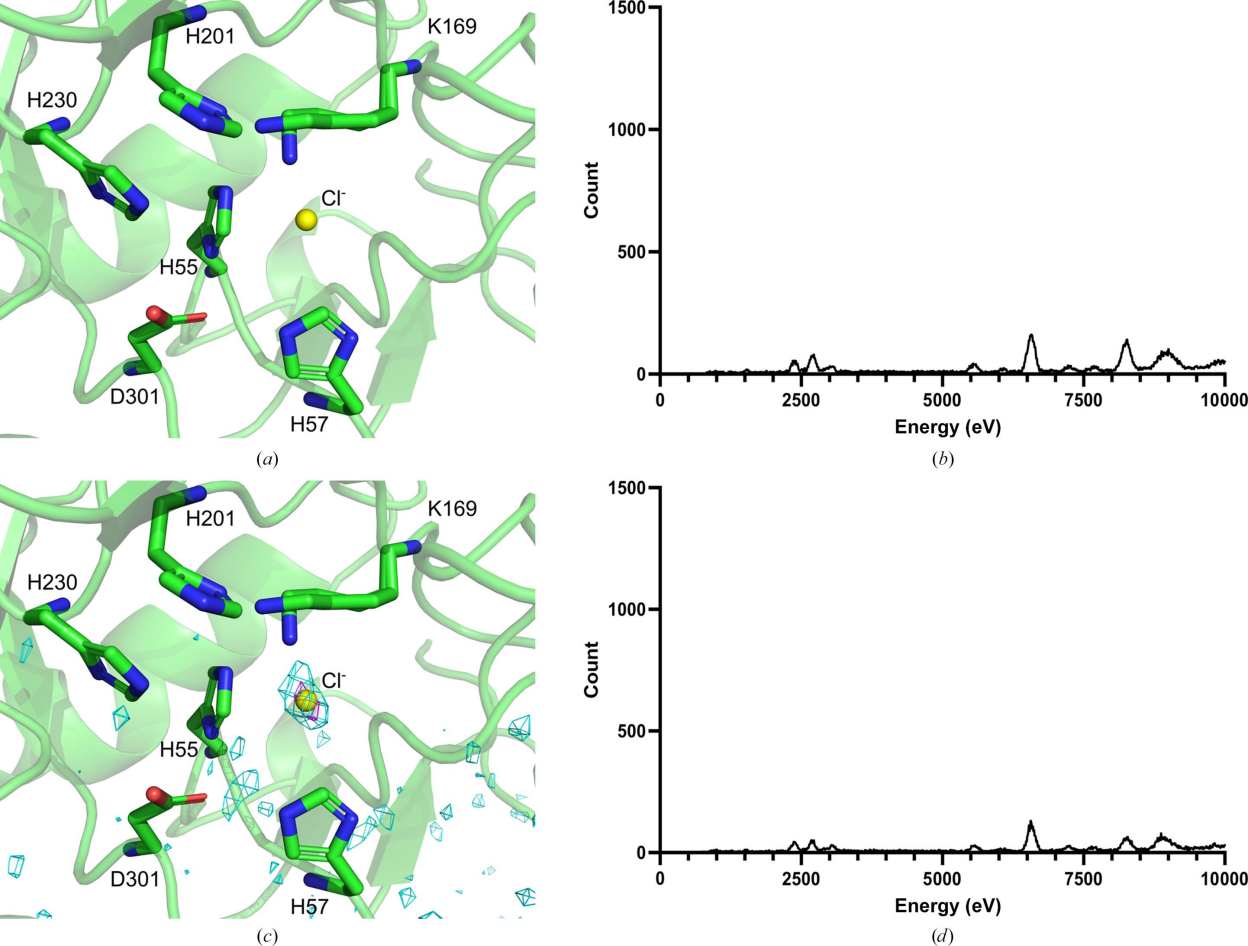
Crystallographic study of apo PTE-R18. (*a*) Structure of the apo PTE-R18 chain *A* active site from data collected at 13 keV (PDB entry 8uqw). (*b*) Fluorescence emission of the apo PTE-R18 crystal used to collect diffraction data at 13 keV. (*c*) Structure of the apo PTE-R18 chain *A* active site from data collected at 9.5 keV (PDB entry 8uqx) with the anomalous map at 3σ (cyan) and 5σ (magenta). (*d*) Fluorescence emission of the apo PTE-R18 crystal used to collect diffraction data at 9.5 keV.

X-ray diffraction data were collected from apo PTE-R18 crystals at 13 and 9.5 keV to maximize the anomalous scattering from Zn(II) and Ln(III) ions, respectively (Table 1 ▸). This was performed to confirm the absence of the metal ions from the active site of apo PTE-R18. Both the 13 keV (1.50 Å resolution; PDB entry 8uqw) and 9.5 keV (1.52 Å resolution; PDB entry 8uqx) structures show a lack of anomalous signal at the expected Zn(II) positions in the active site, indicating the successful removal of Zn(II) and the lack of any anomalous scatterer which could be confused for an Ln(III) signal (Figs. 2 ▸ *a* and 2 ▸ *c*). However, in the 9.5 keV structure an anomalous signal visible to 6σ was present in the active site; this signal was not present in the 13 keV structure above noise. The anomalous signal was suggested to be a result of the Cl *K* edge (2.8 keV) and thus was modelled as a chloride in both structures given the proximity to Lys169 and the abundance of NaCl in the sample from the purification buffers (Merritt, 2012 ▸).

The fluorescence scans performed on the crystals showed a lack of X-ray emission lines at the values expected for Zn(II) (8630, 8615 and 9572 eV), Eu(III) (5845, 5816, 6456, 6843 and 7480 eV) and Gd(III) (6057, 6025, 6713, 7102 and 7785 eV) (Figs. 2 ▸ *b* and 2 ▸ *d*), again indicating the removal of Zn(II) from PTE-R18 and that the crystals contained no Ln(III) (Bearden, 1967 ▸; Kortright & Thompson, 2009 ▸).

In both PDB entries 8uqw and 8uqx the active-site residue Lys169 was modelled in two separate conformations (Figs. 2 ▸ *a* and 2 ▸ *c*); normally Lys169 undergoes spontaneous carboxylation upon metal binding and directly coordinates to the active-site Zn(II) ions (Tokuriki *et al.*, 2012 ▸). However, in the apo structure Lys169 is decarboxylated and thus is capable of adopting two conformations.

In both structures residues 260–275 of chain *A* were unable to be modelled due to a lack of electron density, whereas in chain *B* these residues were modelled into clear density. This loop (loop 7) inherently undergoes conformational fluctuations during catalysis (Jackson *et al.*, 2009 ▸; Caldwell *et al.*, 1991 ▸). Previous directed evolution of wild-type PTE into PTE-R18 resulted in stabilization of loop 7, and loop 7 was able to be built into both chains *A* and *B* of Zn-PTE-R18 crystal structures (Campbell *et al.*, 2016 ▸; Tokuriki *et al.*, 2012 ▸). This suggests that the removal of Zn(II) resulted in increased mobility of loop 7, which was observed in chain *A* of the crystal structure, but loop 7 of chain *B* was rigidified by stabilizing crystal contacts and was able to be modelled.

Previous structures of wild-type PTE demonstrated that the apo PTE crystal structure was substantially different from the crystal structure of the holoenzyme (Benning *et al.*, 1994 ▸, 1995 ▸). In contrast, our apo PTE-R18 structure is very similar to the holoenzyme crystal structures, with r.m.s.d. values of 0.184 and 0.191 Å for PDB entries 8uqw and 8uqx, respectively, against PDB entry 4e3t (Tokuriki *et al.*, 2012 ▸). This suggests that the enzyme structure was stabilized during the course of laboratory directed evolution performed previously, resulting in an apoenzyme crystal structure that is similar to that of the holoenzyme.

### 3.2. Mononuclear lanthanide binding

#### 3.2.1. Ln(III)-PTE-R18 crystallography

**Figure 3:**
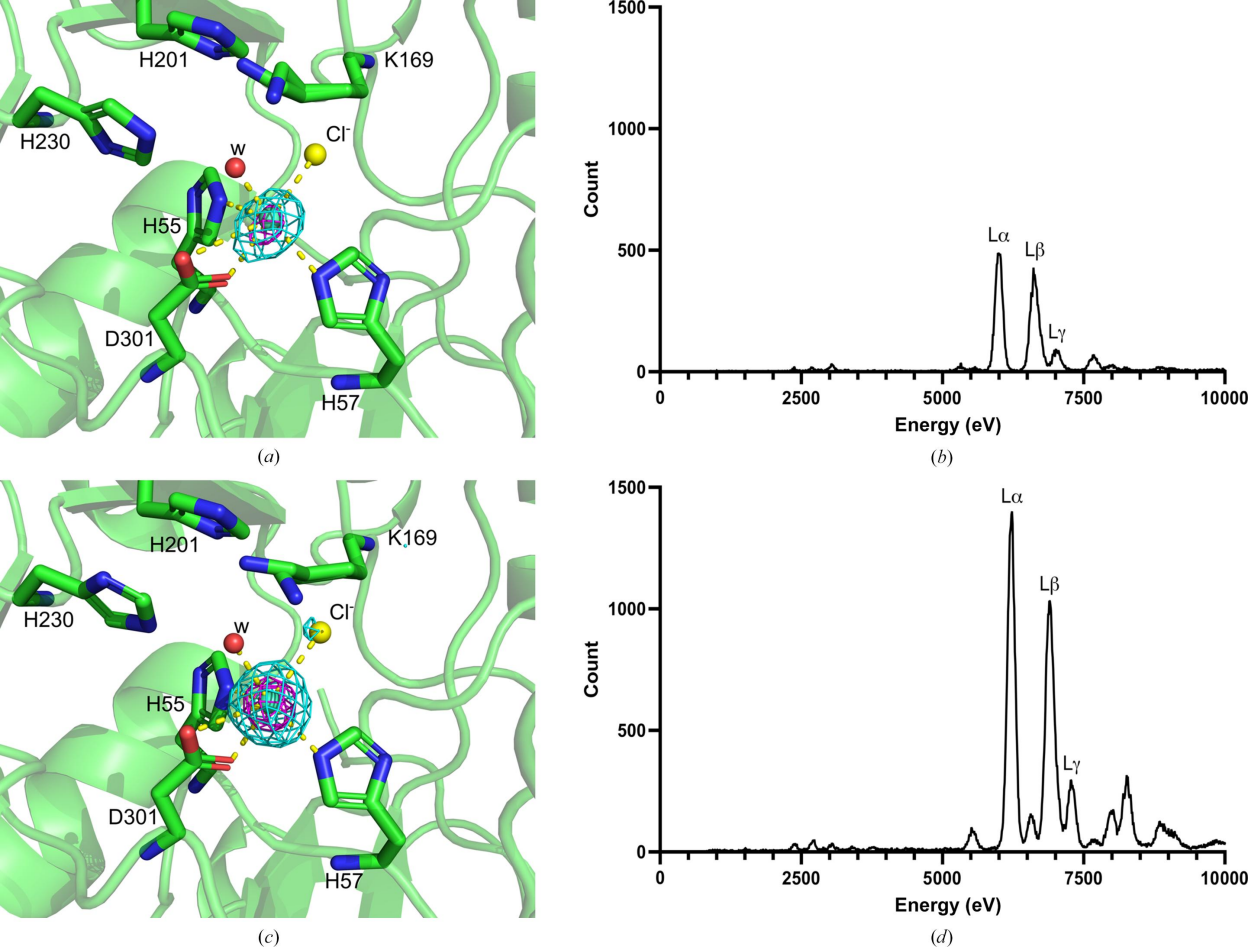
Crystallographic study of Ln(III)-PTE-R18. (*a*) Structure of the Eu-PTE-R18 active site from data collected at 9.5 keV (PDB entry 8uqy) with the anomalous map at 5σ (cyan) and 10σ (magenta). (*b*) Fluorescence emission of the Eu-PTE-R18 crystal. (*c*) Structure of the Gd-PTE-R18 active site from data collected at 9.5 keV (PDB entry 8uqz) with the anomalous map at 4σ (cyan) and 20σ (magenta). (*d*) Fluorescence emission of the Gd-PTE-R18 crystal.

A range of Ln(III) ions were chosen for co-crystallization to provide a variation in radius and Lewis acidity, with La(III), Pr(III), Sm(III), Eu(III) and Gd(III) being co-crystallized with apo PTE-R18. Diffraction data were collected at 9.5 keV from the resulting crystals to maximize the anomalous scattering from any bound Ln(III) ions. The structures of crystals grown with La(III), Pr(III) and Sm(III) were solved to 1.50, 2.16 and 1.84 Å resolution, respectively, and showed a lack of electron density and anomalous signal within the active site, indicating that the desired Ln(III) was not bound within the active site of PTE-R18. Both the Eu(III)-bound (1.78 Å resolution; PDB entry 8uqy) and Gd(III)-bound (1.61 Å resolution; PDB entry 8uqz) structures showed a strong single spherical anomalous signal within the active site, indicating mononuclear binding of Ln(III) to PTE-R18 (Figs. 3 ▸ *a* and 3 ▸ *c*). The greater ionic radius of Eu(III) and Gd(III) compared with Zn(II) would explain the observation of only one ion being accommodated within the active site. These anomalous signals were present to over 12σ and 40σ for Eu(III) and Gd(III), respectively, supporting their placement in the structure. However, these anomalous signals were only present in chain *A* of the PTE-R18 dimer in both structures. Metals were modelled into this density and the occupancies were refined to 16% and 24% for Eu(III) and Gd(III), respectively. These low occupancy values and the presence of the metal in chain *A* only could be the result of weak binding to apo PTE-R18 or due to Ln(III) coordination by abundant solvent molecules, such as the high-concentration cacodylate or MPD, which would sequester Ln(III) from PTE-R18. Furthermore, the presence of the Ln(III) ions in only chain *A* of the dimer could be the result of altered conformations or dynamics between the chains; in chain *A* of the Gd-PTE-R18 structure residues 260–274 (260–275 in Eu-PTE-R18) were unable to be modelled due to a lack of electron density, whereas in chain *B* these residues were modelled; this was also the case for the apo PTE-R18 structures.

We expected both chains *A* and *B* to have bound Ln(III). However, it may be that the binding of Ln(III) to PTE-R18 does not stabilize loop 7 as Zn(II) does, which would explain the missing electron density for loop 7 of chain *A* in the Ln(III)-bound structures. The lack of Ln(III) in chain *B* could be explained as follows: when loop 7 of chain *B* adopts the rigid conformation that is required for crystallization, the monomer forms a conformation that is incapable of coordinating Ln(III), potentially ejecting any large Ln(III) ion from the active site and resulting in chain *B* being metal-free in the crystal. Alternatively, the binding of an Ln(III) ion to chain *A* of PTE-R18 could result in slight conformational changes to chain *B* that make subsequent Ln(III) binding unfavourable, resulting in the binding of Ln(III) ions to only one chain of the dimer.

**Figure 4:**
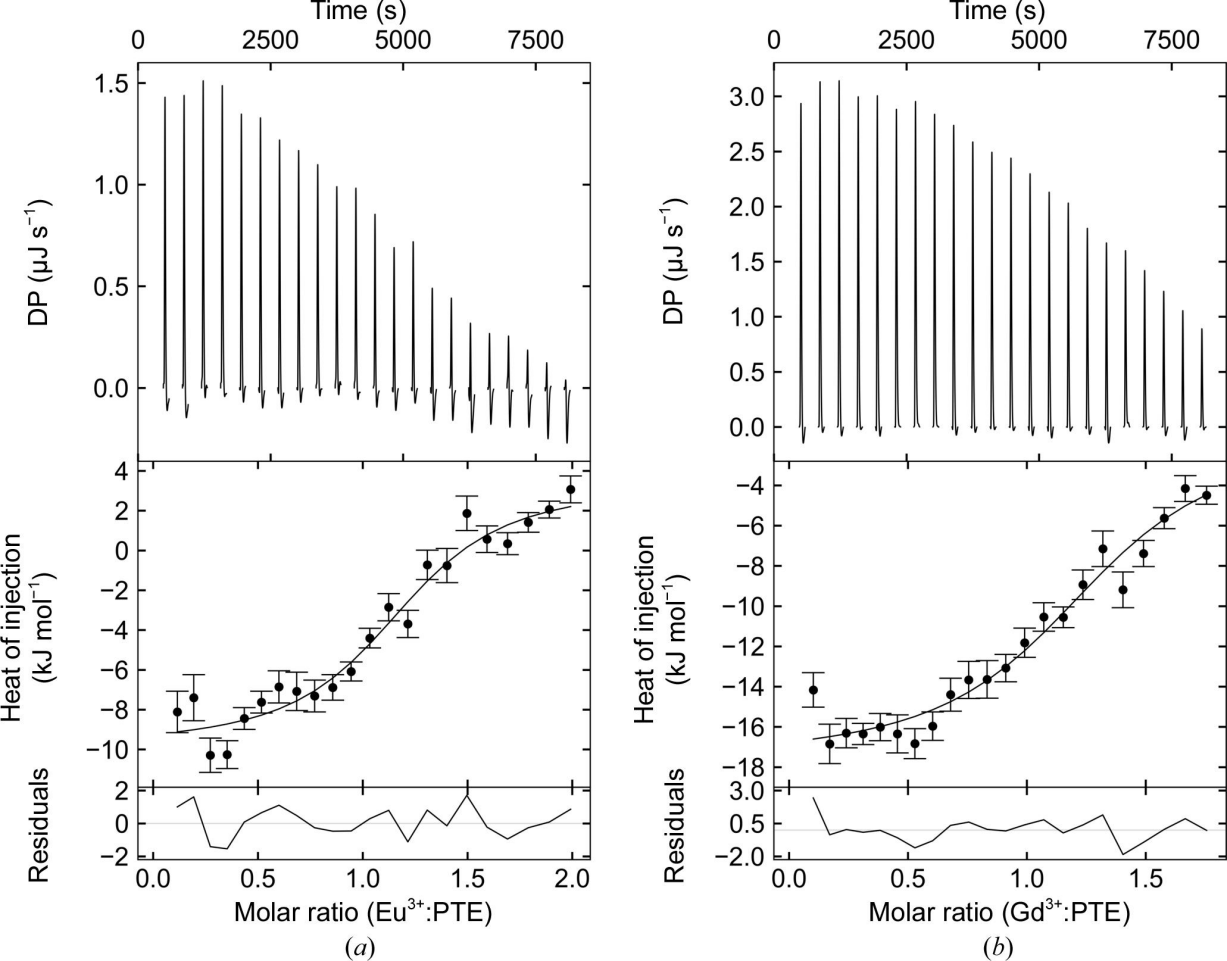
ITC analysis of Ln(III) binding to PTE. ITC data for metal binding to apo PTE-R18 processed using the A + B ↔ AB binding model (*N* = 1). (*a*) Isotherm for EuCl_3_. (*b*) Isotherm for GdCl_3_.

Nonetheless, the fluorescence scans displayed X-ray emission lines at the expected values for Eu(III) and Gd(III) (Figs. 4 ▸ *b* and 4 ▸ *d*), further indicating that Eu(III) and Gd(III) are the coordinating metals (Bearden, 1967 ▸; Kortright & Thompson, 2009 ▸).

The Eu(III)- and Gd(III)-bound structures show identical coordination within the PTE-R18 active site, being coordinated by His55, His57, Asp301, H_2_O (252 in PDB entry 8uqy and 158 in PDB entry 8uqz) and a Cl^−^ bridging from Lys169 (Figs. 3 ▸ *a* and 3 ▸ *c*). The positioning of the Ln(III) ion is near-identical to the α-Zn(II) ion in previous PTE-R18 structures (PDB entries 4e3t, 4gy0 and 4gy1) with very similar coordination, varying by the Ln(III) ions having two coordinate bonds from Asp301 rather than one and by the Ln(III) ions having a bridging Cl^−^ rather than a carboxylated Lys169 (Tokuriki *et al.*, 2012 ▸). This was rather unexpected, as structures of Zn-PTE-R18 show that Lys169 is carboxylated, providing two hard coordinating O atoms; however, this is not the case in the Eu(III)- and Gd(III)-bound structures, despite these metals favouring hard Lewis bases and high coordination numbers. Also, the bridging Cl^−^ would be less entropically favourable when compared with direct amino-acid coordination, but Cl^−^ was present in the apo structures, suggesting that the entropic cost to binding would not be substantial with regard to the Cl^−^ ion.

Interestingly, both Ln(III) ions are coordinated to histidine residues, which has not previously been observed in protein structures, and have a CN of six, with five coordinate bonds from the protein. This low CN, combined with coordination from slightly softer histidines, could be the reason for the low metal occupancy in the structures. Typically, lanthanide-bound protein structures have CNs of 9–10, although some have been reported to be as low as seven, and these coordination groups are hard Lewis bases, often oxygen. Indeed, a *de novo* lanthanide-binding protein has previously been designed by the Zeymer group with four coordinating Glu residues and one water, and an engineered chimeric nuclease from the Franklin group was coordinated using one Glu, three Asp and one Thr residues (Caldwell *et al.*, 2020 ▸; Sirish & Franklin, 2002 ▸; Kim *et al.*, 2001 ▸; Welch *et al.*, 2001 ▸). Furthermore, the natural lanthanide-binding proteins XoxF and LanM have been described to coordinate using two Asp, one Glu and one Asn residues and three groups of the cofactor PQQ, and one Glu, four Asp and one Thr residues, respectively (Cook *et al.*, 2019 ▸; Cotruvo, 2019 ▸; Cotruvo *et al.*, 2018 ▸; Pol *et al.*, 2014 ▸).

These structures illustrate the ability of PTE-R18 to bind Eu(III) and Gd(III) in the active site of one chain of the dimeric enzyme and show that histidines can be ligands for the coordination of metal ions in lanthanoproteins.

#### 3.2.2. Isothermal titration calorimetry

To further substantiate mononuclear binding, and to provide binding-affinity data, isothermal titration calorimetry (ITC) was performed using apo PTE-R18 with EuCl_3_ and GdCl_3_. Both the Eu(III) (*N* ≃ 1.1) and the Gd(III) (*N* ≃ 1.1) titrations suggested mononuclear binding of the metal to each monomer of the apo PTE-R18 dimer (Figs. 4 ▸ *a* and 4 ▸ *b*). This suggests that Eu(III) and Gd(III) do bind to both chains *A* and *B* of PTE-R18 and that the lack of metal in chain *B* is likely to be the result of crystallization. The isotherms also indicated *K*_d_ values of 11 µ*M* (95% confidence interval 7.2–17.2 µ*M*) and 20 µ*M* (95% confidence interval 14.2–26.8 µ*M*) for Eu(III) and Gd(III), respectively. Based upon these dissociation constants and the use of 2.77 m*M* Ln(III) during the crystallization experiments (100 times the *K*_d_), we could expect occupancy values of 90%, rather than 16% and 24%. However, these dissociation constants are based upon solution chemistry and differences would be expected as a result of the crystal contacts.

These *K*_d_ values are relatively high when compared with other lanthanide-binding proteins, which could be a result of the low CN and the relatively weak ligands. For example, Zeymer’s *de novo* lanthanide-binding protein, with numerous hard ligands, was described as having subfemtomolar affinity (Caldwell *et al.*, 2020 ▸). Moreover, the equally numerous and hard ligands of LanM resulted in picomolar affinity values for lanthanides (Cook *et al.*, 2019 ▸; Cotruvo, 2019 ▸; Cotruvo *et al.*, 2018 ▸). Therefore, it could be concluded that the weak binding of the metals was due to the low coordination number and the softness of the histidine ligands. Indeed, EF4 of LanM has an Asn ligand in one position where EF1, EF2 and EF3 have Asp, and this reduces the affinity to micromolar values (Cotruvo *et al.*, 2018 ▸).

This, however, does not rule out Eu-PTE-R18 and Gd-PTE-R18 as potential catalysts, as the Franklin chimeric nucleases were determined to have affinity values for Eu(III) of 3–20 µ*M* whilst being catalytically active (Sirish & Franklin, 2002 ▸; Kim *et al.*, 2001 ▸). Also, the high-affinity Zeymer protein and LanM completely saturate the primary coordination sphere of the metal, leaving no space for a substrate to coordinate; thus, it may not be reasonable to expect such high affinities from a substrate-accessible lanthanoenzyme. Furthermore, these high-affinity proteins use negatively charged ligands, which would reduce the Lewis acidity of the metal, thus reducing a catalytically favourable characteristic of Ln(III).

### 3.3. Esterase activity of Ln(III)-PTE-R18

The coordination of a catalytically relevant metal ion into the active site of PTE-R18 led us to hypothesize that there should be catalytic activity. Given that apo PTE-R18 showed no esterase activity against the more reactive pNPB, we tested for esterase activity against 2NH (Table 2 ▸), which PTE-R18 has been documented to hydrolyse (Tokuriki *et al.*, 2012 ▸).

This kinetic analysis indicated that aqueous Eu(III) and Gd(III) had no detectable esterase activity against 2NH under these conditions, whereas Eu(III)- and Gd(III)-bound PTE-R18 displayed esterase activity against 2NH, as determined by the detection of 2N and hexanoic acid. Both Eu(III)-PTE-R18 and Gd(III)-PTE-R18 showed increased conversion at pH 8.0 compared with pH 7.0, which is likely to be a result of Eu(III) and Gd(III) having p*K*_a_ values of 7.66 and 7.87, respectively, resulting in increased deprotonation of a metal-ion-coordinated water at pH 8.0 to form a stronger nucleophile for attack at the carbonyl of 2NH, consistent with the proposed mechanism of PTE-R18 (Brown & Ekberg, 2016 ▸; Tokuriki *et al.*, 2012 ▸). Thus, despite coordinating a single large metal cation, PTE-R18 was capable of binding 2NH within the active site and the Ln(III) holoenzyme was capable of catalysing ester-bond hydrolysis.

## 4. Conclusion

To conclude, this work demonstrated the ability of PTE-R18 to coordinate a single Eu(III) or Gd(III) ion within the active site and described the first observation of Ln(III) ions being coordinated by histidine residues in proteins. The ability of this enzyme to coordinate catalytically important lanthanide metals into the accessible active site suggests potential for lanthanoenzyme-based catalysis, as was demonstrated by the esterase activity against 2NH. Furthermore, this work provides the opportunity for mutagenesis experiments to increase the CN and Lewis base hardness of coordinating groups, which could result in an altered affinity of PTE-R18 for Eu(III) and Gd(III).

## Supplementary Material

PDB reference: PTE-R18, apo, 13 keV, 8uqw

PDB reference: 9.5 keV, 8uqx

PDB reference: bound to europium(III), 8uqy

PDB reference: bound to gadolinium(III), 8uqz

Supplementary Figures. DOI: 10.1107/S2059798324002316/jb5062sup1.pdf

## Figures and Tables

**Table 1:** Crystallographic statistics Data-collection, processing and refinement statistics for the crystal structures deposited as PDB entries 8uqw, 8uqx, 8uqy and 8uqz. Values in parentheses are for the highest resolution shell.

	Apo PTE-R18	Apo PTE-R18	Eu-PTE-R18	Gd-PTE-R18
PDB code	8uqw	8uqx	8uqy	8uqz
Data collection				
Temperature (K)	100	100	100	100
Diffraction source	MX2, Australian Synchrotron	MX2, Australian Synchrotron	MX2, Australian Synchrotron	MX2, Australian Synchrotron
Wavelength (Å)	0.953	1.305	1.305	1.305
Detector	Dectris EIGER X 16M	Dectris EIGER X 16M	Dectris EIGER X 16M	Dectris EIGER X 16M
Detector distance (mm)	130	130	130	150
Rotation range per image (°)	0.1	0.1	0.1	0.1
Total rotation range (°)	360	360	360	360
Data processing				
Space group	*P*2_1_2_1_2	*P*2_1_2_1_2	*P*2_1_2_1_2	*P*2_1_2_1_2
*a*, *b*, *c* (Å)	85.63, 86.22, 89.16	85.87, 86.50, 89.30	85.51, 85.89, 89.34	85.71, 86.14, 89.25
α, β, γ (°)	90, 90, 90	90, 90, 90	90, 90, 90	90, 90, 90
Mosaicity (°)	0.10	0.11	0.11	0.12
Resolution range (Å)	44.58–1.50 (1.53–1.50)	44.65–1.52 (1.55–1.52)	44.67–1.78 (1.82–1.78)	44.62–1.61 (1.64–1.61)
Unique reflections	105750 (5167)	101871 (4875)	63759 (3593)	84562 (3978)
Completeness (%)	99.7 (99.1)	99.2 (97.1)	100.0 (100.0)	98.1 (95.1)
Multiplicity	40.6 (41.1)	26.1 (26.2)	26.2 (22.2)	40.0 (36.5)
〈*I*/σ(*I*)〉	14.2 (1.3)	15.7 (1.2)	18.7 (1.2)	21.2 (1.7)
CC_1/2_	1.000 (0.665)	0.999 (0.475)	0.999 (0.655)	0.999 (0.662)
*R*_p.i.m._	0.021 (0.673)	0.020 (0.665)	0.022 (0.668)	0.021 (0.968)
*R*_merge_	0.132 (4.314)	0.098 (3.407)	0.109 (3.131)	0.132 (4.232)
*R*_meas_	0.134 (4.367)	0.100 (3.473)	0.111 (3.204)	0.133 (4.291)
Wilson *B* factor (Å^2^)	22.49	26.88	30.73	25.19
Refinement				
Resolution range (Å)	39.60–1.50 (1.55–1.50)	39.68–1.52 (1.57–1.52)	42.94–1.78 (1.84–1.78)	42.86–1.61 (1.66–1.61)
Reflections, working set	105582 (10363)	101760 (9876)	63627 (6240)	84469 (8085)
Reflections, test set	5212 (552)	5062 (440)	3227 (313)	3960 (299)
Final *R*_work_	0.1839 (0.3166)	0.1913 (0.4734)	0.1617 (0.3186)	0.1707 (0.3417)
Final *R*_free_	0.2060 (0.3435)	0.2130 (0.5447)	0.2011 (0.3577)	0.1955 (0.3695)
No. of atoms				
Total	5335	5275	5268	5322
Protein	4932	4916	4936	4953
Ligand	46	46	91	91
Water	385	341	297	334
R.m.s. deviations				
Bond lengths (Å)	0.009	0.006	0.011	0.009
Angles (°)	1.19	0.93	1.32	1.28
Average *B* factors (Å^2^)				
Overall	35.81	38.09	43.36	35.46
Protein	35.35	37.82	42.97	35.03
Ramachandran plot				
Favoured (%)	96.20	96.83	97.01	96.71
Allowed (%)	3.33	2.85	2.67	2.98
Outliers (%)	0.48	0.32	0.31	0.31
Rotamer outliers (%)	0.95	0.77	0.57	0.57
Clashscore	4.60	5.22	4.29	4.57

**Table 2:** Ln(III)-PTE-R18 esterase activity Conversion of 2NH to 2N at pH 7.0 and 8.0 with EuCl_3_ and GdCl_3_ with (+) and without (−) apo PTE-R18.

		2NH:2N	
Ln(III)	pH	+	−
Eu(III)	7.0	60:40	100:0
	8.0	9:91	100:0
Gd(III)	7.0	40:60	100:0
	8.0	25:75	100:0
